# Fidelity monitoring in complex interventions: a case study of the WISE intervention

**DOI:** 10.1186/s13690-018-0292-2

**Published:** 2018-08-29

**Authors:** Taren Swindle, James P. Selig, Julie M. Rutledge, Leanne Whiteside-Mansell, Geoff Curran

**Affiliations:** 1 Department of Family and Preventive Medicine, University of Arkansas for Medical Sciences, 4301 W. Markham St, #530, Little Rock, AR 72205-7199 USA; 2 College of Public Health, University of Arkansas for Medical Sciences, 220 UAMS Campus Dr, #781, Little Rock, AR 72205 USA; 3 College of Applied and Natural Sciences, Louisiana Tech University, 305 Wisteria St, #3167, Ruston, LA 71272 USA; 4 Department of Pharmacy Practice and Psychiatry, University of Arkansas for Medical Sciences, 4301 W. Markham St, #522-4, Little Rock, AR 72205-7199 USA

**Keywords:** Fidelity, Behavioral interventions, Nutrition, Obesity prevention, Implementation science

## Abstract

**Background:**

Researchers face many decisions in developing a measurement tool and protocol for monitoring fidelity to complex interventions. The current study uses data evaluating a nutrition education intervention, Together, We Inspire Smart Eating (WISE), in a preschool setting to explore issues of source, timing, and frequency of fidelity monitoring.

**Methods:**

The overall study from which these data are drawn was a pre/post design with an implementation-focused process evaluation. Between 2013 and 2016, researchers monitored fidelity to evidence-based components of the WISE intervention in 49 classrooms in two Southern states. Data collectors obtained direct assessment of fidelity on a monthly basis in study classrooms. Research staff requested that educators provide indirect assessment on a weekly basis. We used mean comparisons (*t*-tests), correlations (Pearson’s *r*), and scatterplots to compare the direct and indirect assessments.

**Results:**

No mean comparisons were statistically different. Correlations of direct and indirect assessments of the same component for the same month ranged between − 0.51 (*p* = 0.01) and 0.54 (*p* = 0.001). Scatterplots illustrate that negative correlations can be driven by individuals who are over reporting (i.e., self-report bias) and that near zero correlations approximate the ideal situation (i.e., both raters identify high fidelity).

**Conclusion:**

Our findings illustrate that, *on average*, observed and self-reports may seem consistent despite weak correlations and individual cases of extreme over reporting by those implementing the intervention. The nature of the component to which fidelity is being monitored as well as the timing within the context of the intervention are important factors to consider when selecting the type of assessment and frequency of fidelity monitoring.

**Trial registration:**

NCT03075085 Registered 20 February 2017. *Trial registration corresponds to the funding that supported the writing of this manuscript, not the data collection. The original study was not a trial and was collected without registration. However, the data reported here provided foundational preliminary data for the trial.*

## Background

The success of behavioral interventions depends, in part, on the fidelity with which they are delivered. Fidelity is the “degree to which an intervention was implemented as described in the original protocol or as it was intended by the program developers” [1]. Dane and Schneider summarize that fidelity encompasses (1) program adherence, (2) dose of the program delivered, (3) quality of delivery, (4) participant engagement, and (5) differentiation between critical program features [2]. Assessment of fidelity is crucial because failure of interventions to produce desired change in targeted outcomes (e.g., diet, suicide rates, smoking) may be a result of poor implementation delivery rather than a poorly designed program [3–5]. In preparing to measure fidelity, researchers must decide who will provide fidelity ratings on what aspects of fidelity, at what frequency and at what intervals, with what mode of data collection, and with what standard of fidelity in mind [6]. Researchers must also balance psychometric rigor with pragmatic value [7].

There are two main approaches to sourcing information on fidelity: direct assessment (i.e., observer report) and indirect assessment (i.e., self-report) [6]. Direct measures include completion of ratings by trained observers of videotape, audiotape, or direct observation. Indirect measures include self-reports using pencil and paper surveys or technology-based submissions [8]. Direct observation methods are considered to be more valid but are more resource-intensive; self-report methods require less resources but reflect the implementer’s valuable perceptions. [9] Further, despite the psychometric advantages of direct measures, a 2017 review found that researchers use direct and indirect measures equally as often [10].

Few studies have examined the conditions under which direct and indirect measures of fidelity are most appropriate, and few evaluations provide scientific justification for deciding who provides fidelity information. Further, most available comparisons are limited to studies in the mental health field. In these studies, the correspondence between approaches has varied. In some studies, therapists’ self-reported ratings of fidelity to treatment skills and strategies has demonstrated statistically weak relationships with direct measures of fidelity, [11, 12] with individuals reporting higher fidelity for themselves than observers [13]. However, other studies have found a more nuanced relationship. Correspondence between direct and indirect measures has been shown to be stronger for some practices (e.g. practice coverage, client comprehension, homework assignment) than others (e.g., type of exercises completed with client) [14]. Further, despite overestimation of their fidelity, indirect ratings of fidelity by therapists have at least been consistent across time in their correspondence with direct measures [15]. These studies suggest that indirect measures may still provide useful information in some circumstances for understanding variability in implementation. However, researchers have yet to characterize the circumstance under which indirect measures provide this utility. Further, it is unclear if approaches to fidelity measurement in other fields relate to one another in a similar way. For example, WAVES [16] and High 5 [17] were obesity prevention and nutrition promotion interventions delivered in elementary schools. Both of these behavior change efforts collected direct and indirect measures of fidelity; neither completed or planned comparisons between the measures. There could be important differences from the field of mental health given the variety of experience, education, and training levels held by those asked to implement nutrition interventions.

Regardless of the source of information, researchers typically struggle to balance at least three elements to get a valid and stable measure of fidelity: (a) resource constraints, (b) the ideal number of fidelity assessments, and (b) the ideal interval for fidelity assessment [18]. Their decisions might differ by intervention and by the implementation setting. However, guidance is largely lacking. Fidelity measures in mental health studies comparing direct and indirect assessment have ranged widely from one session [11] to every session for a set period of time [15]. Examples of fidelity frequency and intervals for interventions aimed at student behavior change in classrooms range from weekly for 8–10 weeks [19, 20] to once per year [20]. Frequency of collection may also differ across the source of information with self-report being collected more frequently than direct report when both are used within the same study. For example, self-reported fidelity logbooks for each lesson were requested in both the WAVES study [16] and Krachtvoer healthy diet intervention [21]; direct observations were conducted three times per year per school and once per classroom per year in these studies, respectively. Choices may reflect the resource-intensive nature of direct observations and illustrate a lack of standard in the field about how much fidelity observation is adequate.

The unique strengths and weaknesses of approaches and schedules for monitoring fidelity deserve further exploration across a broader range of intervention types and implementer characteristics. To progress toward guidelines that inform researchers’ choices about selection of fidelity measurements, an important first step is to replicate the comparisons of direct and indirect measures that exist in the field of mental health. Additionally, illustration of how the information captured varies across frequently collected intervals and how approaches to analyzing these data inform conclusions could inform other studies. To this end, our primary objective was to compare direct and indirect measures of fidelity to a nutrition promotion curriculum among early educators across time. We highlight the use of three distinct descriptive analytical approaches and the differential conclusions that they suggest.

## Method

### Study design

The current study uses data evaluating a nutrition education intervention, Together, We Inspire Smart Eating (WISE), in a preschool setting to explore issues of source, timing, and frequency of fidelity monitoring. Between 2013 and 2016, researchers monitored fidelity to the WISE intervention in 49 classrooms in two Southern states. Data collectors obtained direct assessment of fidelity on a monthly basis from 100% of study classrooms, which represents 25% of the total possible sample of lessons. Research staff requested that educators provide indirect assessment on a weekly basis. The overall study from which these data are drawn was a pre/post design [22] with an implementation-focused process evaluation [23]. The implementation strategy to support uptake of WISE in this study was standard to the field, including a one-time training and bi-monthly reminder newsletters. Table 1 described these strategies in accordance with Proctor and colleagues [24] and Powell et al. [25] recommendations and definitions for strategy specification. This study did not focus on testing or comparing implementation strategies.

**Table 1:** WISE implementation strategies

Strategy	Definition	Actors	Action	Temporality	Dose	Justification
Make training dynamic	Training was based on maximizing interactive opportunities & reflected adult learning principles	WISE staff	One-time workshop	1–2 weeks before start of school in the fall	6 h	Typical training length for new ECE program, addressed all core components
Remind /Distribute educational materials	Electronic newsletter delivered to each center director to distribute to staff	WISE staff	Bi-monthly newsletter	Approximately every 2 months	3 newsletters	Low-resource support targeted to observed weaknesses noted by data collectors

### Intervention

WISE is a classroom-based nutrition promotion intervention designed to increase children’s exposure to fruits and vegetables (FV) [26]. WISE is a sensory-based nutrition promotion approach that includes 8-units delivered across the school year through weekly food experiences. These units in chronological order focus on: (1) apples, (2) tomatoes, (3) sweet potatoes, (4) bell peppers, (5) carrots, (6) berries, (7) greens, and (8) green beans. Key components of the intervention (i.e., active ingredients; those with a strong evidence base for impacting child diet) were targeted for fidelity monitoring during WISE lessons including: hands-on interactions in small groups [27–34], use of an owl mascot (i.e., Windy Wise) to promote the FV [35–40], and role modeling by educators [28, 41–44]. Additional detail on the intervention is described elsewhere [26].

### Participants

WISE was implemented in 49 classrooms between Fall 2013 and Spring 2016 in three cohorts; 37 classrooms were Head Start; 12 were kindergarten or first grade. Educators in these classrooms who completed fidelity assessments were a majority female (98.1%) with a bachelor’s degree or higher (63.6%). Nearly two-thirds were African-American (62.5%), and just under one-third were White (32.1%). Years of experience ranged between 1 and 41 years (mean = 15.81, SD = 10.24).

### Measures

For both our direct and indirect assessment of fidelity, we followed the steps outlined by Schoenwald and colleagues [6] for fidelity measurement development: (1) identifying relevant components for monitoring (e.g., specificity, necessity, degree of precision), (2) determining who would provide the ratings, (3) obtaining the ratings, and (4) creating a summary score for the ratings. Both measures were designed with pragmatism in mind [24]. That is, we desired psychometrically sound measures that were feasible in the real-world setting. Each key component included two items to assess the quality of delivery aspect of fidelity [1]. Researchers mirrored items on the direct and indirect assessments and averaged items to create a composite variable for each component. See Table 2 for a comparison of the items on the direct and indirect measures as well as how fidelity ratings were defined for trained observers. Table 3 provides means and standard deviations for each component by observer.

**Table 2:** Fidelity items and definitions by component

Component	Item text on direct observation form	Item text on indirect form	Fidelity defined
Use of Mascot	- Uses Windy WISE in activity - Leads the class in the “Whooo tried it?” chant	- Windy Wise visited during or after our lesson. - I felt confident using Windy Wise.	1 – No mention or sight of mascot 2 – Mentions but does not use 3– 1-2 uses of mascot, present during chant 4 – Mascot is integral, used enthusiastically
Role modeling	- Eats the target food. - Makes positive comments about the target food.	- I made positive comments about the food - I tried the food.	1 – Does not eat/comment 2 – Tried with few groups, 1 comment 3 – Tried with most groups, 2 to 3 comments 4 – Tried with all groups, 4 + comments
Hands-on exposure	- Completes activity in prescribed group size. - Involves children in lesson prescribed.	- I did the lesson with groups of 3 to 6 children at a time. - The children participated in this lesson.	1 – Whole group, Teacher led 2 – Half class, Few children have role 3 – Groups of 7–10, Several children have role 4 – Groups of 4–6, All children have role

**Table 3:** Means and standard deviations by source, unit and component

Composite		1	2	3	4	5	6	7	8	Total
Hands-On Exposure	Observer	2.43 (0.97)	2.60 (0.90)	2.49 (1.07)	2.59 (0.87)	2.28 (0.98)	2.44 (1.00)	2.51 (0.99)	2.51 (0.72)	2.58 (0.76)
	Teacher	2.26 (0.45)	2.33 (0.68)	2.29 (0.69)	2.15 (0.58)	2.29 (0.66)	2.36 (0.62)	2.20 (0.50)	2.18 (0.43)	2.75 (0.85)
Use of Mascot	Observer	2.10 (1.06)	2.08 (0.88)	2.60 (1.04)	2.06 (1.12)	2.47 (1.13)	2.71 (1.02)	2.37 (1.02)	2.41 (1.09)	2.23 (0.83)
	Teacher	3.60 (0.66)	3.53 (0.67)	3.55 (0.64)	3.47 (0.71)	3.61 (0.59)	3.52 (0.63)	3.40 (0.87)	3.49 (0.73)	3.29 (0.72)
Role Modeling	Observer	2.65 (0.99)	2.82 (0.96)	2.84 (0.98)	2.59 (0.99)	2.35 (1.14)	3.26 (0.77)	2.51 (1.06)	2.34 (0.89)	2.77 (0.73)
	Teacher*	0.47 (0.47)	0.57 (0.45)	0.43 (0.44)	0.24 (0.31)	0.25 (0.38)	0.18 (0.34)	0.17 (0.30)	0.14 (0.23)	3.72 (0.74)

*These items were assessed with a yes = 1, no = 0 response set

#### Direct assessment

On a monthly basis, trained observers completed a direct observation of a food experience to assess fidelity. Investigators developed the direct assessment with input from multiple project researchers and refined the assessment through initial pilot testing. This assessment is a one-page, 26-item document completed with pencil and paper. Prior to data collection, observers completed a standardized training consisting of an in-person session with instruction on (a) the intent of each item with provision of examples, (b) distinguishing between fidelity ratings, and (c) discrete integration into the classroom setting. Gold-standard observers conducted these trainings. Gold standard observers were two PIs and one RA with greater than 90% inter-rater reliability. After introduction to the forms and instructions, observers coded a video example with the guidance of a gold-standard observer. Next, observers in training coded a second video independently. Thereafter, observers completed pilot field observations with a gold-standard observer. Staff calculated interrater reliability by determining the percentage of items on which observers rated within a narrow margin of error (± 1 on the *same end* of the rating scale) relative to the gold-standard observer. Before observing classrooms independently, each observer was required to exhibit interrater reliability of 85% with a gold-standard observer on 2 occasions. Observers were assessed for training drift near the mid-point of the intervention year; all remained within standards for interrater agreement. Training is typically between 3 and 4 h. Observers (*N* = 14) included undergraduate students of sociology and child development, graduate-level students in nutrition and psychology, and professionals from education and public health.

#### Indirect assessment

Educators self-reported their use of the key components on a 4-point scale (1 = Not at All, 4 = Very Much) on a one-page, paper and pencil, 27-item survey. As is typical for indirect assessments [6], educators did not receive the detailed training that observers received on the meaning and use of the response rating scale. The research team asked educators to complete a self-report fidelity assessment on a weekly basis. Thus, a maximum of 32 self-report fidelity forms per teacher were possible. Compliance with this request was variable. Educators who provided at least one assessment per month received a fidelity score for the month. Educators ranged in submitting fidelity forms; some only submitted for two months while others had at least one submission for all eight months (mean(M) = 5.65, standard deviation(SD) = 1.99). To create comparable scores across this range of compliance, a monthly average self-report score was created. The variability in completed number of assessments suggests varying acceptability of the indirect assessment between teachers.

### Data analyses

All data were analyzed using SPSS (Statistical Package for the Social Sciences) Version 22 [45]. First, we plotted the means for each of the composite and item variables across all 8 units for both direct and indirect assessment. These mean plots allow for visual examination of the difference between direct and indirect ratings, on average. Next, we ran correlations between direct and indirect measures for each composite and item variable for each of the 8 units. Correlations represent how well a score for an individual on the indirect measure would correspond to a score on the corresponding item/composite on for the direct measure. Finally, we used scatterplots to illustrate how scores on the direct measure (x-axis) were reflected on scores on the indirect measure (y-axis) for individual cases for each of the composite and item variables across all 8 units. This yielded 9 mean plots, 72 correlations, and 72 scatterplots. The results presented highlight examples of the unique information provided by each type of statistical tool.

## Results

### Means

**Fig. 1:**
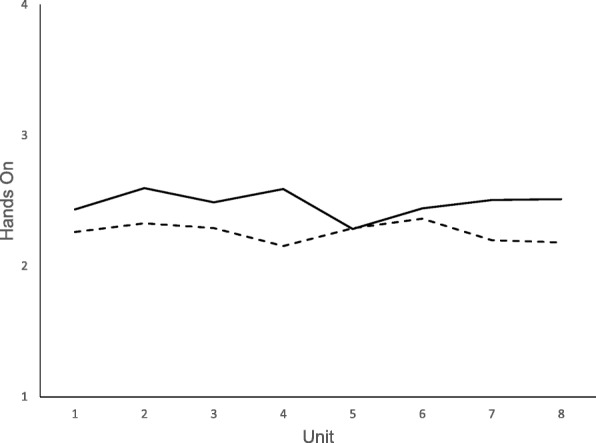
Means Across Units for Direct and Indirect Assessment of Hands On. Legend:

**Fig. 2:**
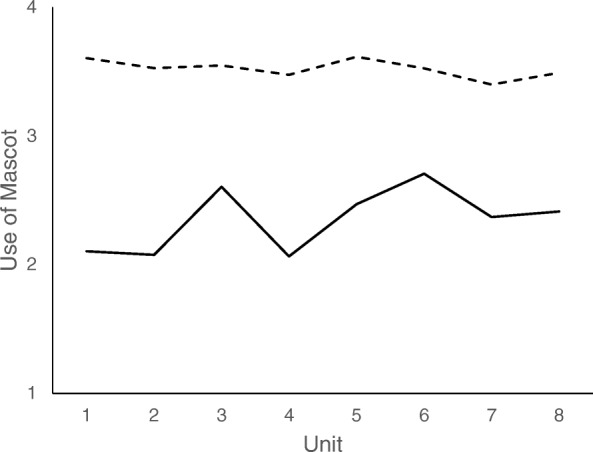
Means Across Units for Direct and Indirect Assessment of Use of Mascot. Legend:

Figure 1 presents the mean plot for Hands On Exposure; and Fig. 2 presents the mean plot for Use of Mascot**.** For all fidelity components, means on the indirect measures were higher (i.e., indicating greater fidelity) than means on the direct measures. The only exception was for the Hands On Exposure fidelity component at one time point (unit 6, See Fig. 1). Fig. 1 demonstrates small differences between the means of direct and indirect assessment of fidelity. The distance between means was small and stable across the school year/unit. Figure 2 demonstrates noticeable gaps between direct and indirect assessments but corresponding dips and peaks in the fidelity reported by each source. The distance between the means decreased across the school year for Use of the Mascot. No means were statistically different.

### Correlations

Correlations between direct and indirect assessment of the Role Modeling composite ranged from a minimum of 0.03 (*p* = 0.87) for unit 7 to a maximum of 0.54 (*p* = 0.001) at unit 5 (See Table 4). Correlations for Use of Mascot composite ranged between − 0.29 (*p* = 0.12) for unit 3 and 0.48 (*p* = 0.02) for unit 8. For 2 of 8 units, negative correlations were observed for Use of Mascot; 4 of 8 correlations were significant. Hands On correlations ranged between − 0.51 at unit 8 (*p* = 0.01) and 0.09 at unit 7 (*p* = 0.68) for the composite variable. The correlations were negative for 7 of 8 units and significant for one unit for Hands On. Thus, although the means appear closer for the Hands On component, correlations between the direct and indirect measures are weaker or more prone to negative direction than for Use of Mascot which had greater differences between the means.

**Table 4:** Correlations between observer and teacher composites by unit

					Unit				
		1	2	3	4	5	6	7	8
Composite	Hands On	−0.24	−0.11	−0.07	−0.36	−0.02	0.09	− 0.23	− 0.51*
	Use of Mascot	0.4	0.46*	−0.29*	0.18	0.24	−0.08	0.44*	0.48*
	Role Model	0.16	0.08	0.43	0.21	0.54*	0.25	0.03	0.23

* *p* < .05

### Scatterplots

**Fig. 3:**
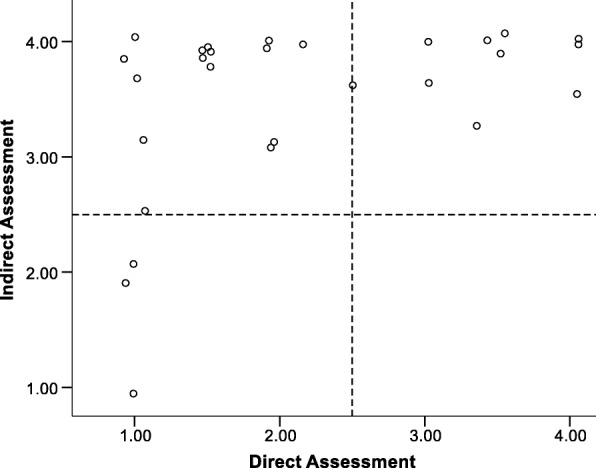
Scatterplot of direct and indirect assessments for use of mascot unit 1

**Fig. 4:**
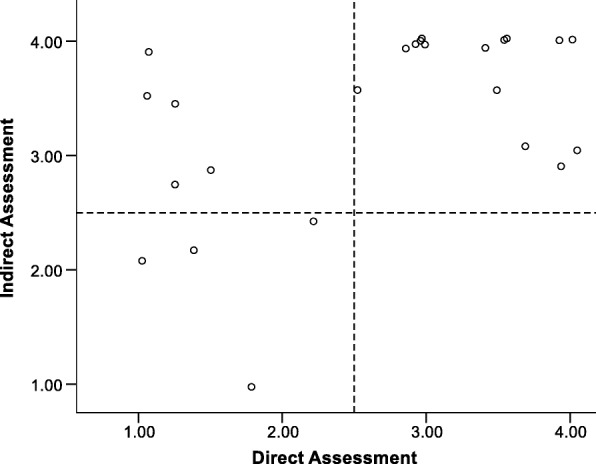
Scatterplot of direct and indirect assessments for use of mascot unit 8

Figure 3 represents the scatterplot for the item of Use of Mascot for unit 1. The corresponding correlation is 0.46 (*p* = 0.02). The mean for the direct assessment was 2.19 (SD = 1.11); the mean for the indirect assessment was 3.61 (SD = .65). Figure 4 represents the same scatterplot for unit 8 [*M*_direct_ = 2.25 (SD = 0.97), *M*_indirect_ = 3.46 (SD = 0.76); *r* = 0.37, *p* = 0.06]. These plots illustrate positive correlations.

The quadrants of the plots represent distinct scenarios. The lower left and upper right quadrants represent consensus between the direct and indirect assessments with those in the lower left agreeing that fidelity is below the mean and those in the upper right agreeing that fidelity is above the mean. The lower right quadrant would reflect cases for which the direct assessment indicated greater fidelity than the indirect self-report measure. The upper left quadrant reflects cases for which the indirect measure indicated higher fidelity than the direct observational measure.

The plots for using Windy at Time 1 and Time 8 (Figs. 3 and 4, respectively) illustrate that direct and indirect assessments had several cases that did improve in their fidelity across the school year (i.e., move to upper right quadrant). There were also several individuals with a continued gap in the accuracy of their reporting on their fidelity across the school year (i.e., did not move to lower left *or* upper right quadrant). However, examination of the means would suggest that the two measures grew closer across the year even though the correlation between the measures decreased.

**Fig. 5:**
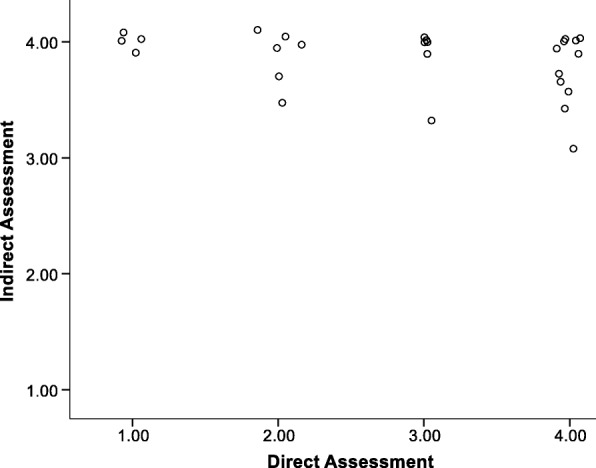
Scatterplot of direct and indirect assessments for child participation unit 1

**Fig. 6:**
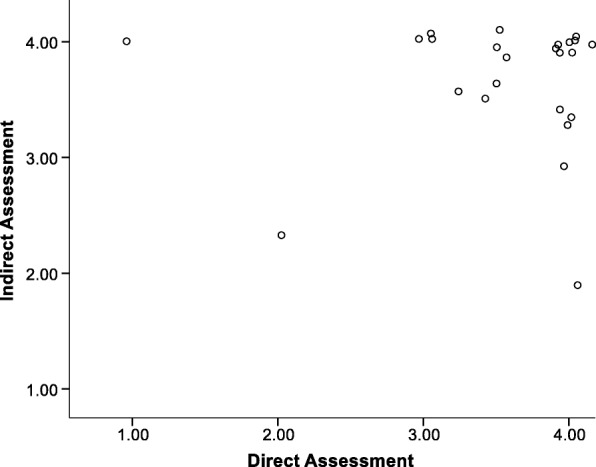
Scatterplot of direct and indirect assessments for child participation unit 8

Figure 5 [*M*_direct_ = 2.88 (SD = 1.11); *M*_indirect_ = 3.85 (SD = 0.28), *r* = − 0.25, *p* = 0.21] and Fig. 6 [*M*_direct_ = 3.55 (SD = 0.73); *M*_indirect_ = 3.66 (SD = 0.56), *r* = 0.06, *p* = 0.78] represent the scatterplots for the item indicating appropriate participation by children at unit 1 and 8, respectively (i.e., one item from the Hands On Exposure component). These plots illustrate the case distribution for a negative correlation (unit 1) and near zero correlation (unit 8) between direct and indirect assessments. At unit 8, the means were closer than at time 1 even though the correlation decreased. The unit 8 plot also illustrates how achieving greater levels of consistent, high fidelity reports across sources (i.e., more cases in upper right quadrant) would result in a near zero correlation between the direct and indirect measures. The shift in cases from the upper left quadrant to the upper right quadrant across the year suggest a true improvement in fidelity which might be attributed to a greater focus of this topic in the newsletters. One case continued to over report at unit 8, which could affect the correlation between measures.

## Discussion

This case study compares direct and indirect assessments of fidelity to a complex intervention for nutrition promotion in educational settings. Our findings illustrate that, *on average*, observed and self-reports may seem consistent despite weak correlations and individual cases of extreme over reporting by those implementing the intervention. These real world data provide an example to help ground future decisions about the “who, what, and when” of fidelity measurements as well as how these data can be analyzed. Few guidelines are available for community-based interventions in making decisions about fidelity measurement. Improvements in standards for fidelity measurement may contribute to reduced numbers of “Type III errors” in which interventions are deemed ineffective due to poor implementation rather than a true ability to produce the desired effect [46].

Consistency in comparisons between direct and indirect assessments in our study differed by the component of fidelity assessed and the time of the school year/intervention. On average, educators in our study reported higher scores than did observers, consistent with the finding that there were cases in the upper left quadrant of scatterplots more often than in the bottom right. This is consistent with previous observation that indirect assessments are prone to self-report bias [6]. In our study, evidence of possible self-report bias was more prevalent (as indicated by cases in the upper left of scatterplots) for some practices than others. Differences in results suggests that self-report bias may be content dependent, reflecting not only a desire to represent oneself well but also a true gap in understanding of how the evidence should be enacted. This is consistent with mental health research in which therapists more accurately reported on use of some techniques than others [14, 15]. In our study, educators made greater shifts toward consistency with observer reports across the school year when there was less subjectivity involved in the ratings (e.g., number of children in group vs. used the puppet enthusiastically). Future research should systematically document traits of evidence-based practices across disciplines on which implementers are better able to rate themselves than others (e.g., concrete versus abstract).

Our case study illustrates frequent collection of both direct and indirect assessments of fidelity across study classrooms. This approach provides a unique opportunity compared to other nutrition intervention studies that typically select a sub-sample of implementers to be assessed at varying intervals (e.g., one lesson per school, three lessons per term per school, 50% of classrooms observed), often using either direct *or* indirect assessment [16, 17, 20, 47]. In our study, both types of fidelity assessment occurred with every unit of the WISE intervention, which coincided, with every month of the school year. On average, educators demonstrated increases in fidelity for some components across time (i.e., involving children as prescribed in hands on activities), decreases to some components across time (i.e., role modeling), and variability likely due to the content of the unit (i.e., higher observed role modeling for berries, less for greens and green beans). In our study, the intervention content was confounded with time of year. Therefore, patterns may reflect calendar effects such as fatigue as the school year draws to the end (e.g., Role Modeling) or distractions from other demands around the time of the holidays (e.g., drop in Mascot Use in December). Researchers cannot assume that improvements due to practice effects are uniform or that a single observed measure of fidelity provides an accurate assessment of the entire intervention period. Decisions about the timing and frequency of fidelity assessment may benefit from aligning resources with the nature of the intervention itself. Researchers should consider content shifts in the intervention (e.g., fruit/vegetable change in WISE) or contextual seasonality effects (e.g., school year) as key variables to inform the measurement schedule. Infrequent or one-time assessment of fidelity may mask the true relations between direct and indirect assessments for some interventions.

This study intentionally illustrated the application of simple analytic techniques that other research teams or leaders in the community (e.g., administrators at schools and hospitals) could use throughout an implementation study with a low burden. Findings illustrate that the type of analysis used to compare direct and indirect assessments can lead to different conclusions. In our data, the means of direct and indirect assessments were closest for the component of Hands On exposure even though correlations were often weak and negative. Additionally, the means for Mascot Use appeared to be tracking together, capturing similar peaks and valleys in fidelity across time despite the gap between the overall means. Examination of scatterplots suggested a more problematic relationship for several individual cases. Thus, interpretation of means and correlations may lead to conclusions that are not true for individuals. Researchers can also appropriately consider this issue by using mixed level models that account for assessments nested in time within the individual and the individual nested within the site [13], although this approach may be less pragmatic for monitoring throughout implementation.

Collecting both direct and indirect assessments of fidelity at key intervention points may be useful to inform implementation strategies. For example, audit and feedback is an evaluation of performance for a set period of time that is given to an implementer verbally, on paper, or electronically [24]. The provision of audit and feedback would differ for a case in the bottom left quadrant (low fidelity by indirect *and* direct assessment) who is aware he/she is not enacting the practice and an individual in the upper left (high indirect fidelity and low direct fidelity) who is reporting he/she is using the practice when observers report otherwise. Cases in the bottom left may not believe the evidence works or may not be motivated to enact the practice. Cases in the upper left may have a misunderstanding about the meaning of the practice or lack the skill to use it. Providing differential feedback to educators in these two scenarios could result in greater shifts to the upper right quadrant. If resources are limited, interventions could collect direct and indirect assessments only until cases are consistently in the upper right quadrant. The joint measurement and comparison of direct and indirect fidelity assessment is a promising application for improving feedback to implementers given previous research showing that fidelity monitoring supports staff retention when used as part of a supportive consultation process [48]. In mental health interventions, practitioners have reported that feedback on their fidelity is helpful to support their learning and practice [48]. Improving the nuance of this feedback through comparison of direct and indirect assessment may prove even more useful.

The present study has both limitations and strengths. First, the resource-intensive nature of direct fidelity assessment limited the size and diversity of our sample to communities in only two locations. This limitation is likely to affect other studies as well [6], and a balance between study size and rigor of evaluation must be considered. Further, as with most fidelity studies, we developed the fidelity measures to reflect the target intervention. This meant that full validation was neither feasible nor possible. For assessing Use of Mascot, item content was not an exact match between the direct and indirect observations. Further, teachers did not receive separate training in use of the fidelity instrument, as did the observers. However, the establishment of interrater reliability for the observed measure and adherence to existing guidelines for fidelity measurement development [6] provide support for the value ofour approach. Future work should consider what aspects of fidelity can be standardized to apply across diverse contexts and interventions. Finally, we did not design this study to capture adaptations to the intervention, conceiving of all departures from our definition of fidelity as equally detrimental and failing to document any potentially appropriate shifts. We made this decision because we conceived of targets of fidelity monitoring in our study as the active ingredients necessary for influencing change. However, Wiltsey-Stirman and colleagues have identified a range of potential adaptations applicable to complex behavioral interventions (e.g., shortening, adding, repeating) and documented that adaptations to psychotherapies were not detrimental in a review of existing studies [49]. Embedding measures to codify adaptations is important for a holistic understanding of how an intervention is implemented [50–52]. Future researchers should consider including measures of adaptation into evaluation plans.

A number of strengths balance these limitations. The research team collected fidelity frequently in all classrooms, a primary strength of the study. The availability of both direct and indirect assessments across the year allowed us to make comparisons at multiple points in time and across all units of the intervention. In addition, the research team designed the fidelity tool to be brief, simple, and specific to the core evidence-based components of WISE. We sought to employ a pragmatic approach which is key to minimizing burden on the implementers and encouraging fidelity monitoring as a routine process [48]. Although use of the tool was variable, teachers did not voice concerns about the one-page assessment.

Researchers have many opportunities for future research in fidelity assessment. When making connections between fidelity and health outcomes, it is unclear if an aggregate measure should be used or if multiple indicators of fidelity across time would be more appropriate for inclusion in statistical models (i.e., an early, middle, and late fidelity score). Resource limitations may prevent multiple measures of fidelity in which case researchers lack guidance on when to time the assessment [53] or model variability in its distance from measurement of the outcomes. Currently, Beidas and colleagues [54] are conducting a randomized control trial to compare the costs and accuracy of three approaches to self-report fidelity measurement (i.e., behavioral rehearsal, chart-stimulated recall, and self-report) in cognitive behavior therapy interventions. Future studies will need to determine if these findings replicate to other content areas in which the implementers and interventions have different characteristics. For example, future research could compare direct and indirect measures after more rigorous training of the implementers on self-assessment or after an initial coaching session comparing an indirect to direct assessment. In our work with WISE specifically, we will seek to determine a minimum level of fidelity that corresponds to significant impacts on various child outcomes. Similarly, we will determine how differently timed fidelity measurements relate to outcomes. Finally, considerations of fidelity measurement source and timing will be important for future studies which seek to test associations between implementation strategies and shifts in fidelity to core components. For example, measurement of fidelity relative to delivery and use of implementation supports (e.g., newsletters in this study) may provide insight into impact of implementations strategies in particular contexts. Further, quality fidelity measures of both the innovation and the implementation intervention will be essential to tease out which strategies contribute to improved implementation [55].

## Conclusions

The National Institute of Mental Health has set a priority of “developing valid and reliable measures of treatment quality and outcomes that can be applied at the person, clinic, system, and population levels” as a key step in improving the quality and equity of outcomes for patients [56]. This study illustrates that the source and timing of the fidelity instrument are important variables to consider for gathering a valid fidelity measure for a specific innovation. Developers of evidence-based interventions should provide guidance on what fidelity information needs to be gathered and if collection of fidelity may be sensitive to different intervals of the intervention itself.

## Abbreviations

ECE: Early childhood educator
FV: Fruits and vegetables
M: Mean
PI: Principal investigator
RA: Research assistant
SD: Standard deviation
SPSS: Statistical package for the social sciences
WISE: We inspire smart eating
